# The Administration of the Willesden Workhouse

**Published:** 1914-07-25

**Authors:** 


### THE ADMINISTRATION OF THE WILLESDEN WORKHOUSE.

The resignation of the master and matron of the Willesden Workhouse has enabled the Board of Guardians to recast entirely the administration of the workhouse. A special committee of the Board reported that the main building was designed for and was essentially an infirmary. Of 604 inmates, 308 were immediately under the care of the medical officer, 248 were old men and women who frequently required medical attention, and only 48 were able-bodied. Having regard to these facts the committee were of opinion that the time had arrived for the appointment of a resident medical officer who should be in charge of the infirmary part of the institution. They recommended that the Local Government Board should be asked to sanction the appointment of a resident medical superintendent of the infirmary who should also be the medical officer of the workhouse, and a steward of the infirmary, acting under the direction of the medical superintendent, to be also master of the workhouse. These recommendations met with some opposition, but were finally adopted by the Board by fourteen votes to five.

We draw special attention to these proposals because we have repeatedly advocated this system of administration in the case of those workhouses where the infirmary cannot be completely separated from the workhouse. If accompanied by the appointment of a matron of the infirmary responsible to the medical superintendent and the Board of Guardians for the nursing arrangements, the system gives to the medical superintendent that complete administrative control of the sick wards which is absolutely essential for the proper treatment of the patients. If sanctioned by the Local Government Board it will form a valuable precedent.

In this connection the correspondence which has recently taken place between the Council of the British Medical Association and the Local Government Board has a special interest. In a letter addressed to that Board, criticising the Poor-Law Institutions Order, 1913, the Council "notes with regret that it is definitely laid down in the Order that the master is to control, subject to the direction of the Guardians, the institution and the officers, including the medical officers. The Association has always emphasised the importance of medical control of medical arrangements in institutions for the sick, and considers that a system which places the supreme control of the sick wards in the hands of a lay officer, and subordinates to him the medical and nursing staff, is radically wrong and is bound to give unsatisfactory results. In view of the openings which the Council believes such a system will afford for bickering and unpleasantness among the officers of such institutions and the consequent lessening of the efficiency of the service, the Council would urge that in the interests of the sick the hospital wards should be entirely separated administratively from the rest of the workhouse, the chief medical officers being placed in control of the medical arrangements."

In reply to this letter the Secretary to the Local Government Board wrote: "With regard to the question of general administrative control I am to point out that the Order in question does not apply to institutions provided wholly for the sick, and that it would not as a rule be practicable in an institution to which the Order applies to sever the administration of the sick wards from the administration of the rest of the institution."

The Willesden proposals will bring home to the Local Government Board that at any rate one important Board of Guardians finds no great difficulty in carrying this separation out.

As a matter of fact there are many other workhouses, at present administered under the Poor-Law Institutions Order, in which a similar administrative reform could be easily introduced. As we pointed out in our number of May 9, in commenting upon a reply of the President of the Local Government Board to a question by Mr. Astor, there are about seventy-five Poor-Law institutions in England and Wales, each containing over 200 beds for sick patients, under the control of the workhouse master. In many of them the infirmary is completely separated structurally from the workhouse, although standing within the workhouse grounds. In their case it is obvious that no difficulty would arise in adding administrative to structural separation, and in those cases where the sick wards are actually within the workhouse the difficulties could be easily overcome. The inferior status and underpayment of the Poor-Law medical officer have led in the past to, and are causing now, an enormous waste in health and in money. The service has presented few attractions to well-qualified and experienced men, and therefore in most Unions the medical work has been organised by men without any special qualifications for the work unaided by expert and experienced advisers. So we see to-day costly infirmaries, equipped in every way to deal with acute cases, filled with senile and chronic cases.

